# The Influence of Pin Deviation on the Fracture Correction and the Fixator Adjustment with Sensitivity and Kinematic Analysis

**DOI:** 10.1155/2018/9267570

**Published:** 2018-10-22

**Authors:** Xia Zhao, Jianfeng Li

**Affiliations:** ^1^Beijing University of Technology, Chaoyang District, Beijing 100124, China; ^2^Beijing University of Technology; Address: No. 100, Pingleyuan, Chaoyang District, Beijing 100124, China

## Abstract

Fracture correction is important orthopedics operation and can be performed by unilateral external fixator. Due to pin deviations being inevitable during the operation, searching the impacts of pin deviation on the fracture correction and fixator joint adjustment are important. This study puts forward evaluation index with sensitivity analysis for investigating the impact of three orientation deviations and three position deviations on fracture correction. Meanwhile, based on a clinical case from a 28-year-old female, the influence of pin deviation on the adjustment of fixator joints was analyzed by inverse kinematic method. Different pin deviations have different sensitivity; the sensitivity of orientation deviation is relatively larger than position deviation. The existence of pin deviation will result in the change of the adjustment value of fixator joints. In addition, the experiments of seven situations were established to verify the analytical results. This study revealed the sensitivity of different pin deviations which can also be used to predict the adjustment value of fixator joints and the accuracy of fracture correction. This research also helps to reduce operation time and decrease damage to soft tissue by reducing the frequency of inserting pins.

## 1. Introduction

Bone fracture is becoming a very prominent research topic mainly due to aging of population and traffic accident [1]. Fracture reduction is usually performed in orthopedics doctors to ensure that fractured bones were restored to their original location. It has been shown that fracture site using mechanical means through external fixators may have beneficial effects on bone healing and remodeling [2–4]. If fracture has not been accurately corrected, fracture mal-union or other fracture dislocation problems can lead to complications, such as early degenerative disease caused by abnormal joint contact pressures [5–7]. Complex types of malalignment often involve pediatric orthopaedic patients and they are often treated by osteotomy [8]. Regulating the rotational and translational joints of the fixator is usually necessary to reduce fracture and correct complex deformities [9, 10]. But the pins with different position deviations and orientation deviation will affect the accuracy of fracture correction. Clinically, the procedure of inserting pins into bone is usually performed artificially before an external fixator is applied for reduction. Under ideal situations, pins are inserted perpendicular to the bone and the fixator is applied in neutral or straight position [11]. However, in the actual situation, the existence of pin deviations is inevitable and it is difficult to insert the pins into the bone accurately and appropriately as ideal situation. Because the fixator has its own limitation, removing the attached fixator, redrilling new pin-holes, and then installing fixator on the new drilled pin-holes are sometimes necessary, but this kind of behavior will be harmful to bone tissue and extend the operation time [12]. If orthopedic surgeon knows how the pin deviation affects fracture correction and the fixator joint regulation rule, then they will be very skilled using external fixator. Meanwhile, if surgeons had more freedom to better arrange the pin-bone relationship and were not distracted to the subsequent reduction again, the quality of operation would be greatly improved.

To the author's knowledge, although many prior studies discussed the sensitivities of two biomechanical methods for fracture healing [13], the adjustability of two unilateral external fixators (Othofixs Dynamic Axial fixator and Dynafixs Standard Tibia fixator) for fracture reduction [14, 15] and the possibility of their nonaxial dynamization after fracture reduction [16], however, where pin deviation has the greatest impact on the accuracy of fracture reduction and the influence of pin deviations on the adjustment values of fixator joint, have been rarely investigated due to lack of a theoretical analysis.

So the aim of this study was, based on a clinical case, to discuss the effect of different pin deviations on fracture correction through sensitivity analysis method, through this study where pin deviation that has the greatest impact on fracture dislocation can be found. Secondly, how to adjust the fixator joints could help correct fracture completely when different pin deviations exist, which was also investigated through inverse kinematic analysis method where joints values can be directly obtained through analytic formulas. The establishment of the experiment also proves the correctness of the analytical results. This is of practical importance since from a physician's standpoint knowing how to accurately correct fracture dislocation is by understanding the relationship between the pin deviations and the adjustment of fixator joints, which can also help to provide theoretical guidance and improve the design of fixator.

## 2. Methods

### 2.1. Kinematic Model

A 6-degree-of-freedom (6-DOF) unilateral external fixator (Dyna-extor, Korean) was used in this study. In this study, only one bone pin at each bone segment is considered for rigid-body in kinematic analysis. This usage method has been reported in a previous article [17].

**Figure 1:**
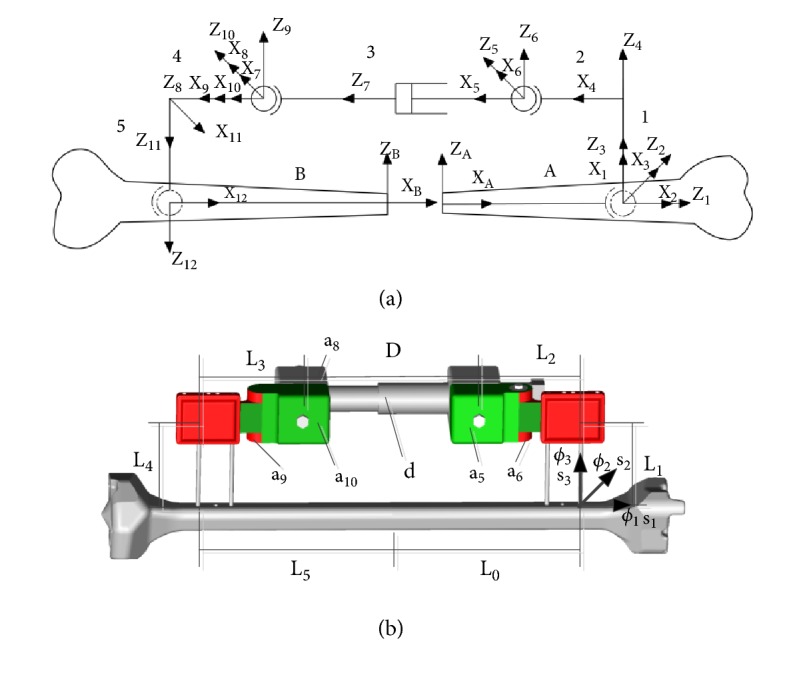
(a) Kinematic model of bone and fixator system, “A”—distal bone; “1”—distal pin; “2”—distal pin clamp; “3”—the telescoping mechanism; “4”—proximal pin clamp; “5”—proximal pin; “B”—proximal bone. (b): The physical model of fixator and bone system, L_0_=L_5_=75mm; L_2_=L_3_=30mm; L_1_=L_4_=65mm; D=90mm; “a_5_, a_6_”— two rotational variables near distal bone, “a_8_  a_9_, a_10_”—three rotational variables near proximal bone; “d”—translational variable; “*φ*_1_, *φ*_2_, *φ*_3_”—angle deviations of pin; “s_1_, s_2_, s_3_”— position deviations of pin.

Taking proximal bone (B) as a reference, supposing distal bone has three Z_1_, Z_2_, Z_3_ axes, having three orientation deviations (*φ*_1_  *φ*_2_  *φ*_3_) and three position deviations (s_1_  s_2_  s_3_), which may exist in the process of inserting pins into bone, shown in Figure 1. Detailed joint parameters of unilateral external fixator are shown in Table 1. The parameters of pin deviation for unilateral external fixator are shown in Table 2.

### 2.2. Establishment of Sensitivity Analysis Model

The precision of fracture reduction is affected by the position deviation and orientation deviation of pin. The effects of different pin deviations on the precision of fracture reduction are different. In order to find out which pin deviation has the greatest impact on the precision of fracture correction, the sensitivity analysis of pin deviation is needed, which describes the effect of degree of the small variation of pin deviations on the fracture correction [18].

The error calculation model of the fixator device can be established, which can be expressed explicitly.

(1) B=M4 AM5 4M6 5M7 6M8 7M9 8M10 9M12 10MB 12=Ms1,s2,s3,φ1,φ2,φ3

B is the continuous differentiable function of *s*_1_, *s*_2_, *s*_3_, *φ*_1_, *φ*_2_, *φ*_3_*;* the first-order Taylor series expansion of (1) can be expressed as follows:

(2) $$\begin{array}{c} M\left(s_{1}+\Delta s_{1},s_{2}+\Delta s_{2},s_{3}+\Delta s_{3},\varphi_{1}+\Delta \varphi_{1},\varphi_{2}+\Delta \varphi_{2},\varphi_{3}+\Delta \varphi_{3}\right)=M\left(s_{1},s_{2},s_{3},\varphi_{1},\varphi_{2},\varphi_{3}\right)+\frac{\partial M}{\partial s_{1}}\Delta s_{1}+\frac{\partial M}{\partial s_{2}}\Delta s_{2}+\frac{\partial M}{\partial s_{3}}\Delta s_{3}+\frac{\partial M}{\partial \varphi_{1}}\Delta \varphi_{1}+\frac{\partial M}{\partial \varphi_{2}}\Delta \varphi_{2}+\frac{\partial M}{\partial \varphi_{3}}\Delta \varphi_{3}+O\sqrt{{\left(\Delta s_{1}\right)}^{2}+{\left(\Delta s_{2}\right)}^{2}+{\left(\Delta s_{3}\right)}^{2}+{\left(\Delta \varphi_{1}\right)}^{2}+{\left(\Delta \varphi_{2}\right)}^{2}+{\left(\Delta \varphi_{3}\right)}^{2}} \end{array}$$

In (2), Δ*s*_1_, Δ*s*_2_, Δ*s*_3_, Δ*φ*_1_, Δ*φ*_2_, Δ*φ*_3_ are the small amount of the *s*_1_, *s*_2_, *s*_3_, *φ*_1_, *φ*_2_, *φ*_3_; removing the high order terms, the following formula can be obtained.

(3) $$\begin{array}{c} M\left(s_{1}+\Delta s_{1},s_{2}+\Delta s_{2},s_{3}+\Delta s_{3},\varphi_{1}+\Delta \varphi_{1},\varphi_{2}+\Delta \varphi_{2},\varphi_{3}+\Delta \varphi_{3}\right)=M\left(s_{1},s_{2},s_{3},\varphi_{1},\varphi_{2},\varphi_{3}\right)+\frac{\partial M}{\partial s_{1}}\Delta s_{1}+\frac{\partial M}{\partial s_{2}}\Delta s_{2}+\frac{\partial M}{\partial s_{3}}\Delta s_{3}+\frac{\partial M}{\partial \varphi_{1}}\Delta \varphi_{1}+\frac{\partial M}{\partial \varphi_{2}}\Delta \varphi_{2}+\frac{\partial M}{\partial \varphi_{3}}\Delta \varphi_{3}=B+\Delta B \end{array}$$

And then the sensitive matrix of pin deviations can be obtained as follows; in (4), *J* is error sensitivity matrix or error transfer matrix.

(4) $$\begin{array}{c} \Delta M=\Delta B=\frac{\partial M}{\partial s_{1}}\Delta s_{1}+\frac{\partial M}{\partial s_{2}}\Delta s_{2}+\frac{\partial M}{\partial s_{3}}\Delta s_{3}+\frac{\partial M}{\partial \varphi_{1}}\Delta \varphi_{1}+\frac{\partial M}{\partial \varphi_{2}}\Delta \varphi_{2}+\frac{\partial M}{\partial \varphi_{3}}\Delta \varphi_{3}=J\left(\Delta s_{1},\Delta s_{2},\Delta s_{3},\Delta \varphi_{1},\Delta \varphi_{2},\Delta \varphi_{3}\right) \end{array}$$

Inverse kinematic analysis method was adopted to solve *J*. In the mathematical analysis of the kinematic chain, _ _^*A*^*M*_*B*_ is the transformation matrix from the proximal bone segment to the distal bone segment which could be expressed as follows:

(5) MB A=M4 AM5 4M6 5M8 7M9 8M10 9M12 10MB 12=nxoxaxpxnyoyaypynzozazpz0001

__^*A*^ *M*_4_ refers to homogeneous transformation matrix from distal bone to distal pin.^4^*M*_5_ and ^5^*M*_6_ are the rotational matrix of distal revolute joint, ^6^*M*_7_ denotes the translational matrix of the telescoping mechanism, and ^7^*M*_8_  ^8^*M*_9_  ^9^*M*_10_ are the rotational matrix of proximal ball joint. ^10^*M*_12_ expresses matrix from proximal pin to proximal bone and ^12^*M*_B_ represents displacement transformation matrix from the proximal pin to fracture site.

Inverse kinematic analysis method was adopted to solve J. Firstly, multiply [^12^*M*_*B*_]^−1^ [^10^*M*_12_]^−1^ [^9^*M*_10_]^−1^ on both right sides of (5) and obtain (6). [^12^*M*_*B*_]^−1^ [^10^*M*_12_]^−1^ [^9^*M*_10_]^−1^ are the inverse matrix of [^12^*M*_*B*_] [^10^*M*_12_] [^9^*M*_10_], respectively.

(6) MB AMB 12 −1M12 10−1M10 9−1=M4 AM5 4M6 5M7 6M8 7M9 8

(7) H=M4 AM5 4M6 5M7 6M8 7M9 8=H11H12H13H14H21H22H23H24H31H32H33H34H41H42H43H44

(8) M=MB AMB 12−1M12 10−1M10 9−1=M11M12M13M14M21M22M23M24M31M32M33M34M41M42M43M44

Equation (7) denotes the transformation matrix from the coordinate system Z_A_ to Z_9_ in a counterclockwise direction; (8) refers to transformation matrix from the coordinate system of Z_A_ to Z_9_ in a clockwise direction in Figure 1(a). Given that the equations on both sides of (6) are equal, *H* and *M* are equal and first derivatives of *H* and *M* are equal, so (9)-(10) is derived.

(9) $$\begin{array}{c} H_{14}=M_{14}\\ H_{24}=M_{24}\\ H_{34}=M_{34} \end{array}$$

According to (9), a_5_, a_6_, d, can be solved. Detailed process can be shown in appendix.

(10) $$\begin{array}{c} H_{14}^{\prime }=M_{14}^{\prime }\\ H_{24}^{\prime }=M_{24}^{\prime }\\ H_{34}^{\prime }=M_{34}^{\prime } \end{array}$$

According to (10), (11) can be obtained as follows:

(11) $$\begin{array}{c} A_{1}\varphi_{1}^{\prime }+A_{2}\varphi_{2}^{\prime }+A_{3}\varphi_{3}^{\prime }+A_{4}s_{3}^{\prime }-A_{5}s_{2}^{\prime }=Bs_{1}^{\prime }+A_{6}Bs_{3}^{\prime }+A_{7}B\varphi_{2}^{\prime }-A_{8}B\varphi_{3}^{\prime }\\ B_{1}\varphi_{1}^{\prime }+B_{2}\varphi_{2}^{\prime }+B_{3}\varphi_{3}^{\prime }+B_{4}s_{2}^{\prime }+B_{5}s_{3}^{\prime }=B_{6}B\varphi_{1}^{\prime }+B_{7}B\varphi_{2}^{\prime }+B_{8}B\varphi_{3}^{\prime }+B_{9}Bs_{2}^{\prime }-B_{10}Bs_{3}^{\prime }\\ s_{1}^{\prime }-C_{1}\varphi_{2}^{\prime }+C_{2}\varphi_{3}^{\prime }-C_{3}s_{3}^{\prime }=C_{4}B\varphi^{\prime }-C_{5}B\varphi_{2}^{\prime }+C_{6}B\varphi_{3}^{\prime }+C_{7}Bs_{2}^{\prime }+C_{8}Bs_{3}^{\prime } \end{array}$$

Multiplying both sides of (5) by [^14^M_B_]^−1^[^10^M_14_]^−1^[^9^M_10_]^−1^[^8^M_9_]^−1^ [^7^M_8_]^−1^[^6^M_7_]^−1^, (12) is obtained as follows.

(12) M4 AM5 4M6 5=MB AMB 14−1M14 10−1M10 9−1M9 8−1M8 7−1M7 6−1

(13) P=M4 AM5 4M6 5=P11P12P13P14P21P22P23P24P31P32P33P34P41P42P43P44

(14) K=MB AMB 14−1M14 10−1M10 9−1M9 8−1M8 7−1M7 6−1=K11K12K13K14K21K22K23K24K31K32K33K34K41K42K43K44

P denotes the transformation matrix from Z_A_ to Z_6_ in a counterclockwise direction; K refers to the transformation matrix from Z_A_ to Z_6_ in a clockwise direction, as shown in Figure 1(a). *P* and *K* are equal; thus, first derivatives of K and P are also equal, so (14) is derived.

(15) $$\begin{array}{c} K_{14}=P_{14}\\ K_{24}=P_{24}\\ K_{34}=P_{34}\\ K_{13}=P_{13} \end{array}$$

According to (15), a_8_, a_9_, a_10_ can be solved. Detailed process can be expressed in appendix.

(16) $$\begin{array}{c} K_{14}^{\prime }=P_{14}^{\prime }\\ K_{24}^{\prime }=P_{24}^{\prime }\\ K_{34}^{\prime }=P_{34}^{\prime } \end{array}$$

According to (16), (17) can be obtained as follows:

(17) $$\begin{array}{c} D_{1}B\varphi_{2}^{\prime }+D_{2}B\varphi_{3}^{\prime }+Bs_{1}^{\prime }+D_{3}Bs_{3}^{\prime }=D_{4}\varphi_{1}^{\prime }+D_{5}\varphi_{2}^{\prime }+D_{6}\varphi_{3}^{\prime }-D_{7}s_{2}^{\prime }+D_{8}s_{3}^{\prime }\\ E_{1}B\varphi_{1}^{\prime }+E_{2}B\varphi_{2}^{\prime }+E_{3}B\varphi_{3}^{\prime }+E_{4}Bs_{2}^{\prime }-E_{5}Bs_{3}^{\prime }=E_{6}\varphi_{1}^{\prime }-E_{7}\varphi_{2}^{\prime }+E_{8}\varphi_{3}^{\prime }+E_{9}s_{2}^{\prime }+E_{10}s_{3}^{\prime }\\ F_{1}B\varphi_{1}^{\prime }-F_{2}B\varphi_{2}^{\prime }+F_{3}B\varphi_{3}^{\prime }+F_{4}Bs_{2}^{\prime }+F_{5}Bs_{3}^{\prime }=F_{6}\varphi_{2}^{\prime }+F_{7}\varphi_{3}^{\prime }+s_{1}^{\prime }-F_{8}s_{3}^{\prime } \end{array}$$

According to (11) and (17), (18) can be obtained as follows:

(18) $$\begin{array}{c} \left[\begin{array}{cccccc} A_{1} & A_{2} & A_{3} & 0 & -A_{5} & A_{4}\\ B_{1} & B_{2} & B_{3} & 0 & B_{4} & B_{5}\\ 0 & -C_{1} & C_{2} & 1 & 0 & -C_{3}\\ D_{4} & D_{5} & D_{6} & 0 & -D_{7} & D_{8}\\ E_{6} & -E_{7} & E_{8} & 0 & E_{9} & E_{10}\\ 0 & F_{6} & F_{7} & 1 & 0 & -F_{8} \end{array}\right]\times \left[\begin{array}{c} \varphi_{1}\\ \varphi_{2}\\ \varphi_{3}\\ s_{1}\\ s_{2}\\ s_{3} \end{array}\right]=\left[\begin{array}{cccccc} 0 & A_{7} & -A_{8} & 1 & 0 & A_{6}\\ B_{6} & B_{7} & B_{8} & 0 & B_{9} & -B_{10}\\ C_{4} & -C_{5} & C_{6} & 0 & C_{7} & C_{8}\\ 0 & D_{1} & D_{2} & 1 & 0 & D_{3}\\ E_{1} & E_{2} & E_{3} & 0 & E_{4} & E_{5}\\ F_{1} & -F_{2} & F_{3} & 0 & F_{4} & F_{5} \end{array}\right]\times \left[\begin{array}{c} B\varphi_{1}\\ B\varphi_{2}\\ B\varphi_{3}\\ Bs_{1}\\ Bs_{2}\\ Bs_{3} \end{array}\right] \end{array}$$

According to (18), (19) can be obtained as follows:

(19) $$\begin{array}{c} {\left[\begin{array}{cccccc} 0 & A_{7} & -A_{8} & 1 & 0 & A_{6}\\ B_{6} & B_{7} & B_{8} & 0 & B_{9} & -B_{10}\\ C_{4} & -C_{5} & C_{6} & 0 & C_{7} & C_{8}\\ 0 & D_{1} & D_{2} & 1 & 0 & D_{3}\\ E_{1} & E_{2} & E_{3} & 0 & E_{4} & E_{5}\\ F_{1} & -F_{2} & F_{3} & 0 & F_{4} & F_{5} \end{array}\right]}^{-1}\left[\begin{array}{cccccc} A_{1} & A_{2} & A_{3} & 0 & -A_{5} & A_{4}\\ B_{1} & B_{2} & B_{3} & 0 & B_{4} & B_{5}\\ 0 & -C_{1} & C_{2} & 1 & 0 & -C_{3}\\ D_{4} & D_{5} & D_{6} & 0 & -D_{7} & D_{8}\\ E_{6} & -E_{7} & E_{8} & 0 & E_{9} & E_{10}\\ 0 & F_{6} & F_{7} & 1 & 0 & -F_{8} \end{array}\right]\times \left[\begin{array}{c} \varphi_{1}\\ \varphi_{2}\\ \varphi_{3}\\ s_{1}\\ s_{2}\\ s_{3} \end{array}\right]=\left[\begin{array}{c} B\varphi_{1}\\ B\varphi_{2}\\ B\varphi_{3}\\ Bs_{1}\\ Bs_{2}\\ Bs_{3} \end{array}\right] \end{array}$$

Error transfer matrix can be obtained as follows:

(20) $$\begin{array}{c} J={\left[\begin{array}{cccccc} 0 & A_{7} & -A_{8} & 1 & 0 & A_{6}\\ B_{6} & B_{7} & B_{8} & 0 & B_{9} & -B_{10}\\ C_{4} & -C_{5} & C_{6} & 0 & C_{7} & C_{8}\\ 0 & D_{1} & D_{2} & 1 & 0 & D_{3}\\ E_{1} & E_{2} & E_{3} & 0 & E_{4} & E_{5}\\ F_{1} & -F_{2} & F_{3} & 0 & F_{4} & F_{5} \end{array}\right]}^{-1}\left[\begin{array}{cccccc} A_{1} & A_{2} & A_{3} & 0 & -A_{5} & A_{4}\\ B_{1} & B_{2} & B_{3} & 0 & B_{4} & B_{5}\\ 0 & -C_{1} & C_{2} & 1 & 0 & -C_{3}\\ D_{4} & D_{5} & D_{6} & 0 & -D_{7} & D_{8}\\ E_{6} & -E_{7} & E_{8} & 0 & E_{9} & E_{10}\\ 0 & F_{6} & F_{7} & 1 & 0 & -F_{8} \end{array}\right] \end{array}$$

The detailed solution process and concrete expressions of A_1_ ~A_8_, B_1_ ~B_10_, C_1_ ~C_8_, D_1_ ~D_8_, E_1_ ~E_10_, F_1_ ~F_8_ in (11) and (17)~(20) can be explained in the appendix.

### 2.3. Sensitivity Evaluation Index of Pin Deviation

The influence degree of pin deviation on the fracture correction can be expressed by the error sensitivity coefficient. The corresponding amount of various types of deviation of pins is shown in Table 3.

**Figure 2:**
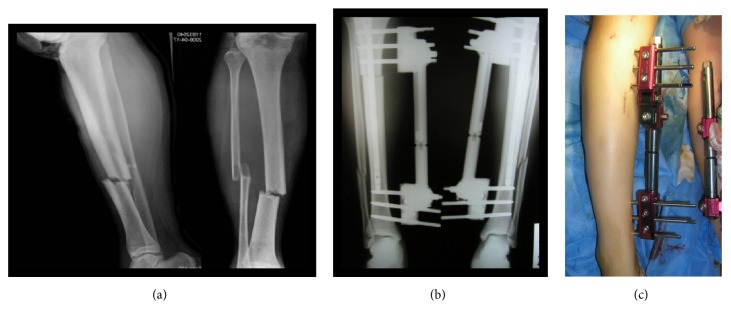
A clinical case. (a) Preoperative X-ray film for a case of tibia deformity. (b) Postoperative anteroposterior X-ray film of limb. (c) Photo showing the limb after application of the unilateral external fixator.

In (21), *qs* is displacement deviation at the fracture site; *q*_*φ*_ is angular misalignment at the fracture site shown as Figure 2. *Js* is error transfer matrix of position deviation of pin; *Jφ* is error transfer matrix of orientation deviation of pin.

(21) $$\begin{array}{c} qs=Js\delta s\\ q_{\varphi }=J_{\varphi }\delta_{\varphi } \end{array}$$

According to (21), using Lagrange operator to establish Lagrange equation,

(22) $$\begin{array}{c} Ls=\delta s^{T}Js^{T}Js\delta s-\lambda s\left(\delta s^{T}\delta s-1\right)\\ L_{\varphi }={\delta_{\varphi }}^{T}J_{\varphi }^{T}J_{\varphi }\delta_{\varphi }-\lambda_{\varphi }\left({\delta_{\varphi }}^{T}\delta_{\varphi }-1\right) \end{array}$$

In (22), *λs* and *λ*_*φ*_ are Lagrange multipliers. According to (22), construct the extreme value of Lagrange conditional equation:

(23) $$\begin{array}{c} \frac{\partial Ls}{\partial \delta s}=0:Js^{T}Js\delta s-\lambda s\delta s=0\\ \frac{\partial L\varphi }{\partial \delta \varphi }=0:J\varphi^{T}J\varphi \delta \varphi -\lambda \varphi \delta \varphi =0 \end{array}$$

In (23), Lagrange multipliers of *λs* and *λ*_*φ*_ are the eigenvalues of *Js*^*T*^*Js* and *J*_*φ*_^*T*^*J*_*φ*_, respectively. The square of displacement deviation q*s* and angular misalignment q*φ* are shown as follows:

(24) $$\begin{array}{c} {\left\|qs\right\|}^{2}=\Delta qs^{T}\Delta qs=\delta s^{T}Js^{T}Js\delta s=\delta s^{T}\lambda s\delta s=\lambda s\\ {\left\|q_{\varphi }\right\|}^{2}=\Delta {q_{\varphi }}^{T}\Delta q_{\varphi }={\delta_{\varphi }}^{T}{J_{\varphi }}^{T}J_{\varphi }\delta_{\varphi }={\delta_{\varphi }}^{T}\lambda_{\varphi }\delta_{\varphi }=\lambda_{\varphi } \end{array}$$

The extreme values of Δ*Bs* and Δ*Bφ* are the root of *λs* and *λ*_*φ*_, respectively:

(25) $$\begin{array}{c} \left\|qs\right\|=\sqrt{\lambda s}=\sqrt{J_{s}^{T}J_{s}}\\ \left\|q_{\varphi }\right\|=\sqrt{\lambda_{\varphi }}=\sqrt{J_{\varphi }^{T}J_{\varphi }} \end{array}$$

Δ*Bs* and Δ*Bφ* have three extreme components, respectively, which can be written as follows:

(26) $$\begin{array}{c} fs_{1}=\sqrt{\lambda s_{1}}=\sqrt{J_{s_{1}}^{T}J_{s_{1}}}\\ fs_{2}=\sqrt{\lambda s_{2}}=\sqrt{J_{s_{2}}^{T}J_{s_{2}}}\\ fs_{3}=\sqrt{\lambda s_{3}}=\sqrt{J_{s_{3}}^{T}J_{s_{3}}} \end{array}$$

In (26), *λs*_1_, *λs*_2_, and *λs*_3_ are the sensitivity coefficient of displacement dislocation Δ*Bs* at the fracture site in X_A_, Y_A_, Z_A_, three directions, respectively, caused by position deviation of pin.

(27) $$\begin{array}{c} {f_{\varphi }}_{1}=\sqrt{{\lambda_{\varphi }}_{1}}=\sqrt{J_{\varphi_{1}}^{T}{J_{\varphi }}_{1}}\\ f_{\varphi 2}=\sqrt{\lambda_{\varphi 2}}=\sqrt{J_{\varphi_{2}}^{T}{J_{\varphi }}_{2}}\\ f_{\varphi 3}=\sqrt{\lambda_{\varphi 3}}=\sqrt{J_{\varphi_{3}}^{T}{J_{\varphi }}_{3}} \end{array}$$

In (25), *λ*_*φ*1_, *λ*_*φ*2_, *λ*_*φ*3_ are the sensitivity coefficient of angular misalignment Δ*Bφ* at the fracture site in three directions of X_A_, Y_A_, Z_A_, respectively, which caused orientation deviation of pin.

Take *λs* and *λ*_*φ*_ as sensitivity evaluation index to assess the impact of position deviation of pin and orientation deviation of pin on the displacement dislocation and angular dislocation, respectively.

### 2.4. Clinical Application

In order to better study the effect of pin deviation on fixator joint adjustment and verify the sensitivity of pin deviation, a case was used.

In clinical practice, 28-year-old female was injured in a road traffic accident and treated with a unilateral external fixator. A 28-year-old female motorcyclist was brought into hospital after having been hit by a car from her right side. She had no medical history, took no regular medication, and had no allergies. The patient provided written informed consent for publication. The case has AP radiograph showing tibia deformity of 20° internal rotation and 20° external rotation and has 15° angular deformity and 5mm displacement deviations in three planes. By adjusting the fixator joints of a_5_, a_6_, d, a_10_, a_9_, and a_8_ with -6°, 20.8°, 9.3mm, -10.6°, -3.3°, and -12° respectively, the fracture deformity can be completely corrected.

Follow-up continued for a mean of 18 months (10–37 months). On the second day after surgery all patients had radiographs (anteroposterior, lateral, and mortise views) (Figure 2(a)). Partial weight bearing was allowed six weeks postoperatively and was gradually increased according to clinical and radiological evidence of union up to full weight bearing after complete union (Figure 2(b)) at a mean of 12 weeks (10–16 weeks). X-ray examination was repeated every month for a period of six months to evaluate bone union and fracture consolidation. Removal of the unilateral fixator was done for all cases after complete union at a mean period of 14 weeks (12–17 weeks). Removal of the fixator was followed by muscle strengthening exercises and physiotherapy.

## 3. Results

In this section, we discuss the sensitivities of different pin deviations. Meanwhile, based on a clinical case, the effect of pin deviation on fracture correction is also analyzed, which can verify the sensitivity of pin deviation from an analytical point of view. Moreover, the experiment was also established to verify the analytic results.

### 3.1. Simulation Results of Pin Deviations Sensitivity

#### 3.1.1. Results of Sensitivity Analysis for Single Pin Deviation

We first investigate the sensitivity of single pin deviation in the appropriate range. The bigger the sensitivity is, the greater the effect of the pin deviation on the precision of the fracture reduction is.

**Figure 3:**
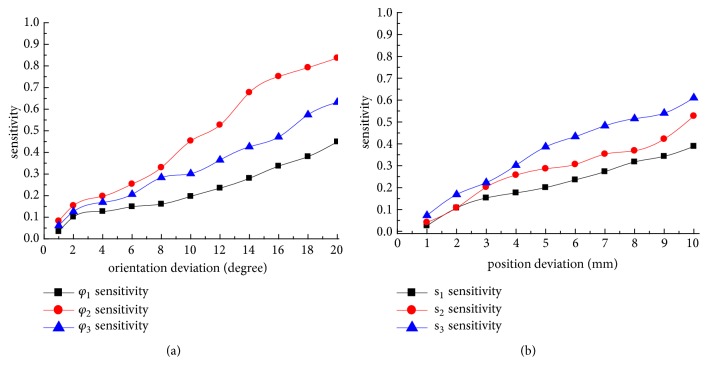
The sensitivity of single pin deviation. (a) The sensitivity of orientation deviation of single pin. (b) The sensitivity of positional deviation of single pin.

Figure 3(a) shows sensitivity curves of three single orientation deviations; it can be noted that *φ*_2_ has the largest sensitivity when compared with *φ*_3_ and *φ*_1_. Besides *φ*_2_ and *φ*_3_ in the entire range of pin deviations, the sensitivity was relatively larger than *φ*_1_. This indicates that residual fracture deformity of B*φ*_2_ is most likely to appear. The possibility of fracture deformity of B*φ*_1_ caused by *φ*_1_ is minimal. Meanwhile, with the increase of the pin deviation, the sensitivity of all pin deviations present an increasing trend; this indicates that the increase in pin deviation may lead to increased sensitivity for fracture dislocations.

From Figure 3(b), it can be seen that the sensitivity of s_3_ is higher than that of s_1_ and s_2_.With the increase of the position deviation, the sensitivity also displays an increasing trend.

The sensitivity of s_1_ is minimal, meaning that it has the least impact on fracture displacement dislocation of Bs_1_, and Bs_3_ has the biggest influence in displacement dislocation due to s_3_ having the biggest sensitivity compared with s_2_ and s_1_.

Comparing Figures 3(a) and 3(b), the maximum sensitivity is *φ*_2_. Orientation deviation of *φ*_3_, *φ*_2_, and *φ*_1_ is relatively larger than position deviation of s_3_, s_2_, and s_1_, respectively. These sensitivity results show that angular dislocation is more likely to occur than displacement dislocation caused by pin deviation, which imply that the orientation deviation has a greater impact on the accuracy of fracture correction than the position deviation and which may be needed to adjust more joints with more amount to ensure fracture correction completely.

In order to investigate whether the sensitivity of double pin deviations has the same regularity, the following sensitivity simulation was performed.

#### 3.1.2. Results of Sensitivity Analysis for Double Deviations

**Figure 4:**
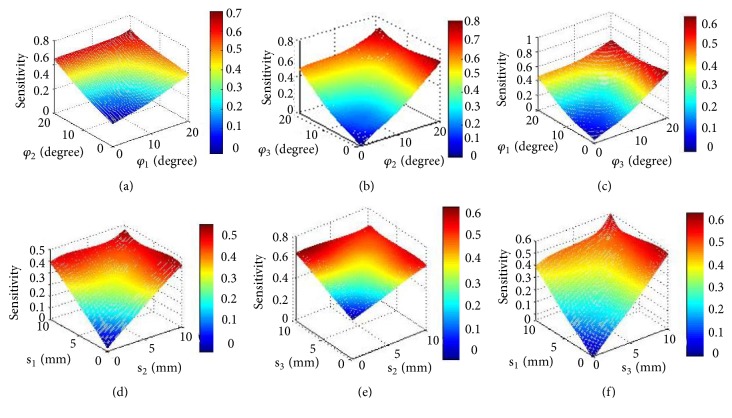
The sensitivity of position and orientation deviation of double deviations of pin. (a), (b), and (c) The sensitivity of position deviation of double deviations of pin. (d), (e), and (f) The sensitivity of orientation deviation of double deviations of pin.

The sensitivity results from Figure 4 help operation doctor to seek the place of maximum sensitivity when there are two kinds of pin deviations simultaneously, as well as predict the change of the fixator's regulation. In Figure 4(a), the sensitivity peak is 0.6986 at *φ*_1_=20°, *φ*_2_=20°; from Figures 4(b) and 4(c), the maximum sensitivity is 0.7618 and 0.6103; they appear in (*φ*_3_=20°  *φ*_2_=20°) and (*φ*_3_=20°  *φ*_1_=20°), respectively. By comparing these three values, it is revealed that the sensitivity of *φ*_2_ is greater than *φ*_3_ and *φ*_1_, because due to existing pin deviation of *φ*_2_, the sensitivity values of 0.6986 and 0.7618 are bigger than 0.6103. Meanwhile, under the condition of having *φ*_2_, the value of 0.7618 is bigger than 0.6986, indicating the sensitivity of *φ*_3_ is higher than that of *φ*_1_. Besides, from Figure 4(a), the higher sensitivity of 0.612 also appears in the (*φ*_2_=20°  *φ*_1_=0°); this illustrates again that *φ*_2_ has a greater influence on fracture correction than *φ*_1_, because the larger deviation sensitivity tends to occur to the side of *φ*_2_. For Figure 4(b), the bigger sensitivity is at (*φ*_3_=0°  *φ*_2_=20°), which is 0.7618, and for Figure 4(c) the bigger sensitivity that is 0.561 appears at (*φ*_3_=20°  *φ*_1_=0°). These results also illustrated that *φ*_2_ has larger impact than *φ*_3_ and *φ*_1_ for fracture dislocation. *φ*_3_ has a greater influence on fracture deformity correction than *φ*_1_.

For Figures 4(d), 4(e), and 4(f), the maximum sensitivity values exist in s_1_=10mm, s_2_=10mm; s_3_=10mm, s_2_=10mm; s_1_=10mm, s_3_=10mm; and they are 0.45437, 0.6371, and 0.59342, respectively. By comparing these three values, the conclusions same as Figure 3(b) can be obtained as follows: the sensitivity of s_3_, s_2_, and s_1_ shows a gradual increasing trend. In Figure 4(d), the higher sensitive value appears at the s_2_=0mm, s_1_=10mm and s_1_=0mm, s_2_=10mm; they are 0.38 and 0.4218, respectively. Obviously, the impact of s_2_ on fracture displacement correction is greater than s_1_. For Figures 4(e) and 4(f), the higher sensitivity values exist at (s_3_=10mm s_2_=0mm) and (s_1_=0mm s_3_=10mm); they all incline the side of s_3_; this indicates that the sensitivity of s_3_ is bigger than s_1_ and s_2_.

So we can put forward a hypothesis; as long as we know the maximum sensitivity of single deviation, the maximum sensitivity of multiple deviations can be found. In order to verify this hypothesis, the following section analyzes the sensitivity of the multiple deviations of pin.

#### 3.1.3. Results of Sensitivity Analysis for Multiple Deviations of Pin

**Figure 5:**
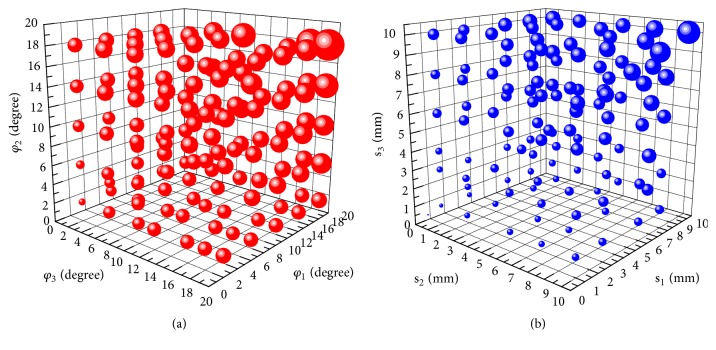
The sensitivity of multiple deviations of pin. (a) The sensitivity of multiple position deviations of pin. (b) The sensitivity of multiple orientation deviations of pin.

For Figure 5, the volume of blue ball and red ball indicates the sensitivity of orientation deviation and position sensitivity, which have three different orientation deviations (*φ*_1_  *φ*_2_  *φ*_3_) and three different position deviations (s_1_  s_2_  s_3_) at the same time, respectively. With the increase of deviation, the volume of the ball increases gradually, so the sensitivity is also increased. For Figure 5(a), the growth rate of the spherical volume in the *φ*_2_ direction was significantly larger than that in *φ*_1_ and *φ*_3_ directions. The change rate in the direction of *φ*_1_ is the smallest. For Figure 5(b), the growth rate of the spherical volume in s_3_ direction was relatively bigger than that in s_2_ and s_1_ directions. The change rate in the direction of s_1_ is also the smallest as the same results in Figures 4(d)–4(f).

Comparing Figure 5(a) with Figure 5(b), the maximum position sensitivity appears in *φ*_1_=20°, *φ*_2_=20°, *φ*_3_=20° and s_1_=10mm, s_2_=10mm, s_3_=10mm, respectively; the results from this figure are consistent with previous assumptions since the maximum sensitivity also appears in the point which is also the maximum sensitivity in the single deviation.

Because the accuracy of fracture correction is guaranteed by adjusting fixator joints, it is necessary to investigate how to adjust fixator that can compensate fracture dislocations caused by pin deviation.

### 3.2. Results of the Effect of Pin Deviations on the Fixator Adjustment

Based on a clinical case in 2.4 (20° internal rotation, 20° external rotation and has 15° angular deformity, and 5mm displacement deviations on three planes). The effect of pin deviations on the fixator adjustment was analyzed.

#### 3.2.1. The Effect of Orientation Pin Deviation on Fixator Adjustment

Under the condition of ideal situation—having no pin deviation, a_5_  a_6_ d a_10_  a_9_  a_8_ can be obtained by (9) and (15), they are -5.99°, 20.8024°, 9.3537mm, -10.5515°, -3.2705°, and -12.1301°, respectively. In the same way, when we have pin deviations, fixator joints also can be solved. Next we investigate the effect of pin deviation on fixator's joints as follows.

**Figure 6:**
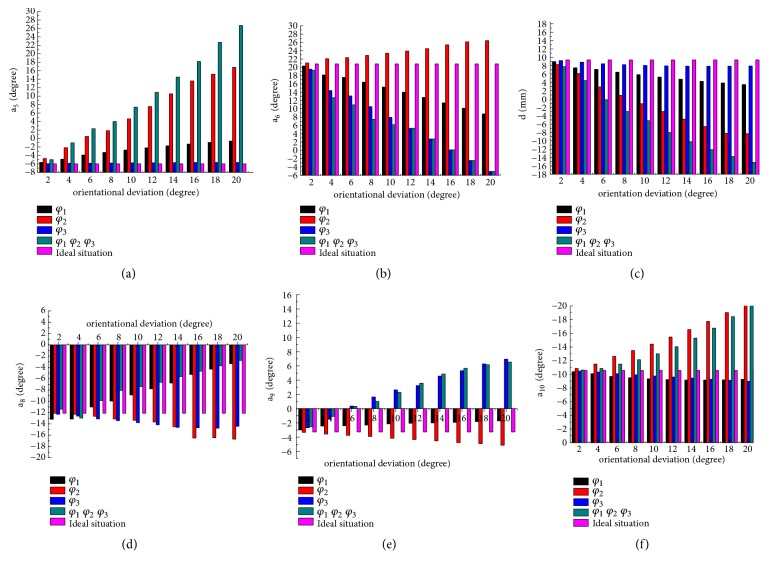
The influence of orientation deviation on the fixator joint adjustment. (a) The influence of orientation deviation on the adjustment of the fixator joint -a_5_. (b) The influence of orientation deviation on the adjustment of the fixator joint a_6_. (c) The influence of orientation deviation on the adjustment of the fixator joint d. (d) The influence of orientation deviation on the adjustment of the fixator joint a_8_. (e) The influence of orientation deviation on the adjustment of the fixator joint a_9_. (f) The influence of orientation deviation on the adjustment of the fixator joint a_10_.

Figure 6 displays the bar charts of the effect of different orientation deviation on the fixator joint adjustment (a_5_  a_6_ d a_8_  a_9_  a_10_). According to the results listed in Figures 6(a)–6(f), the results can be observed that the increase of orientation deviation of *φ*_1_ will lead to rapid decrease of fixator joints of a_8_. Since the regulating range of a_8_ is only 36°, the existence of *φ*_1_ can help a_8_ avoid reaching the maximum value of regulation and expand the solution domain of the fixator.

It can be shown that the adjustment joint of a_5_ is mainly affected by *φ*_2_ and a_5_ and a_10_ need to adjust greater quantity to ensure fracture correction accurately when compared with the condition of having no deviation, but this sharp increase in a_5_ and a_10_ will result in fracture correction incompletely, because the adjustment range of the fixator is limited. On the contrary, the existence of *φ*_2_ can make d decrease a lot; this will expand solution domain of d.

Similarly, with the increase of *φ*_3_, to ensure the fracture was corrected completely, the adjustment amount of a_6_ needs to decrease greatly and a_9_ will reduce a certain value, while *φ*_3_ did not cause a lot of change to other joints compared with ideal situation.

The greater the sensitivity of pin deviation, the greater the adjustment amount of the fixator. According to the above results that *φ*_2_ affects more joints with greater value than *φ*_3_ and *φ*_1_, it can be also proven that the sensitivity of *φ*_2_ is larger than *φ*_3_ and *φ*_1_; nevertheless the sensitivity of *φ*_1_ is minimal.

In addition to this, an important property was found in Figures 6(a)–6(f); when there are more deviations at the same time, the adjustment value of the joint is equal to the sum of the adjustment value of each deviation.

#### 3.2.2. The Effect of Position Deviation of Pin on the Fixator Joint Adjustment

**Figure 7:**
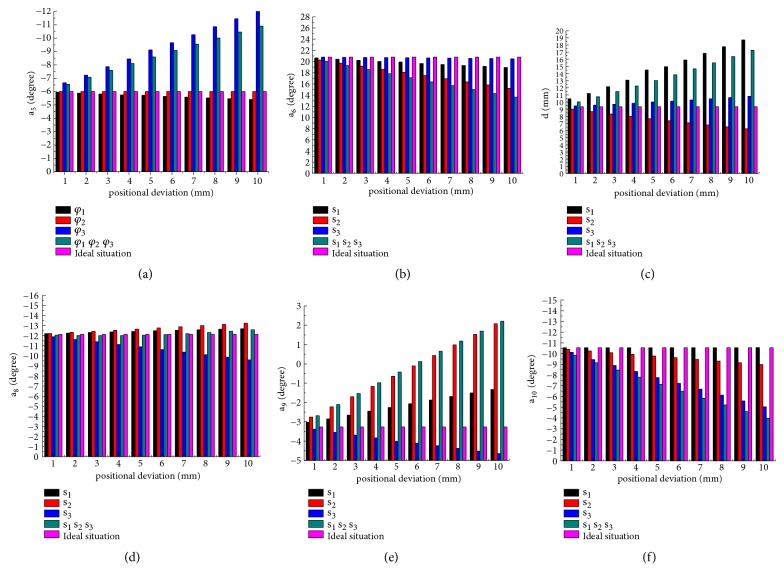
The influence of position deviation on the fixator joint adjustment. (a) The influence of position deviation on the adjustment of fixator joint a_5_. (b) The influence of position deviation on the adjustment of fixator joint a_6_. (c) The influence of position deviation on the adjustment of fixator joint d. (d) The influence of position deviation on the adjustment of fixator joint a_8_. (e) The influence of position deviation on the adjustment of fixator joint a_9_. (f) The influence of position deviation on the adjustment of fixator joint a_10_.

For Figures 7(a)–7(f), the effects of different position deviation on fixator adjustment are presented. As the sensitivity analysis above, there are more joints affected by s_3_; with the increase of s_3_, the joint regulation of a_5_ increased significantly, a_10_ declined with a rapid rate, but a_8_ and a_9_ show a slightly downward trend. The appearance of s_3_ leads a_5_ to reach the limit value in advance and also cause a great residual fracture dislocation and then will delay fracture healing.

When the deviation of s_2_ increases, a_9_ generated a higher regulatory value, a_6_ and d had lower value compared with ideal situation; the existence of s_2_ also makes the fixator joint a_9_ not capable enough to correct deformity which need greater regulation value.

The presence of the position deviation of s_1_ makes d increase significantly and also bring little increase in a_9_ and little decrease in a_5_. Similar to previous position sensitivity analysis, it is conceivable that s_3_ has impact on more fixator joints with high value; the effect of s_1_ on fixator joint is minimal. It also can be found that the influence of pin deviation on the fixator's regulation has superposition characteristic, meaning that just knowing the influence of each pin deviation on adjustment amount of fixator joints and accordingly how to adjust fixator can also be predicted when there are many deviations; the adjustment value of fixator is the sum of adjustment value of every pin deviation.

### 3.3. Experimental Verification

**Figure 8:**
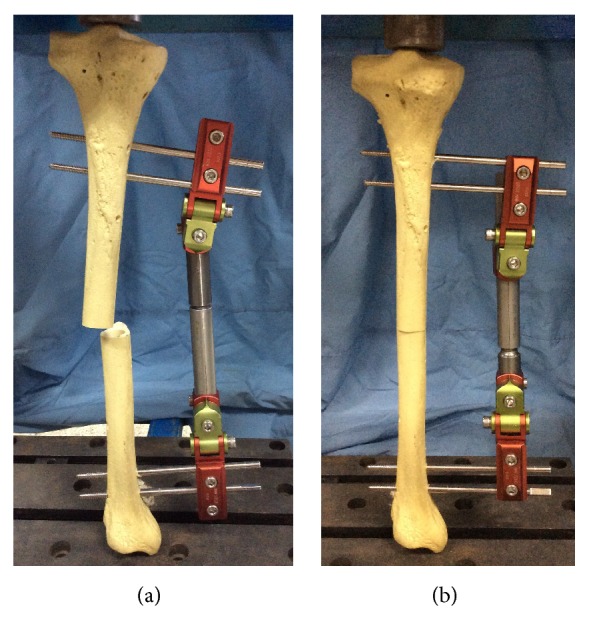
The experiment of fixator-bone system for bone deformity at ideal situation. (a) The experiment of fixator-bone system before deformity correction. (b) The experiment of fixator-bone system after deformity correction.

The experiment of fixator-bone system for deformity correction at ideal situation shown in Figure 8 was established, in order to better verify the analytical results. In experiment, the bone deformation has 20° internal rotation, 20° external rotation, 15° angular deformity, and 5mm displacement deviations on three planes, which is the same as the clinical case. The adjustment values of fixator joints which need to correct deformity are also in accordance with clinical practice, meaning the experiment is reasonable. Moreover, seven situations were also established to verify the analytical results; experimental results are shown in Table 4. The parameters of fixator-bone system in experiment are consistent with analytical model in Figure 1. In the process of experiment, one side of bone was fixed; by adjusting fixator joints, the deformity can be completely corrected.

Table 4 shows the adjustment values of fixator joints at seven situations in experiment. Take the ideal situation as reference, when compared with pin deviations of *φ*_2_ and *φ*_3_, the adjustment value of the fixator joint of a_8_ has the biggest discrepancy between the ideal situation and the existence of pin deviation *φ*_1_. The presence of *φ*_2_ causes a greater change than *φ*_1_ and *φ*_3_ in the amount of a_5_, a_8_, and a_10_. Compared with *φ*_1_ and *φ*_2_, *φ*_3_ has the biggest effect on a_6_ and a_9_. In summary, when compared with ideal situation, the existence of pin deviation will affect the regulating amount of fixator joint, and *φ*_2_ will affect more joints and a greater amount of adjustment is needed; *φ*_1_ has only bigger influences on one joint of a_8_ and *φ*_3_ causes bigger effect on two joints of a_6_ and a_9_. That also indicates that *φ*_2_ has the bigger sensitivity than *φ*_1_ and *φ*_3_, meaning *φ*_2_ has the biggest effect on the fixator joint adjustment. In addition, *φ*_1_ has the minimum sensitivity compared to *φ*_2_ and *φ*_3_. These experimental results are consistent with analytical results for angular deviations.

For positional deviations, take the ideal situation with no pin deviations as a reference; compared with s_2_ and s_3_, the existence of s_1_ makes a bigger change in fixator joint d. Meanwhile, s_2_ has a great effect on a_9_ and a_6_. The existence of s_3_ makes a great contribution to the bigger changes on a_5_, a_8_, and a_10_ than s_1_ and s_2_. These results indicate that s_3_ has the biggest sensitivity on the fixator joint adjustment and s_1_ has minimum sensitivity, which can also verify the correctness of the analytical results.

Table 5 shows the discrepancy between experiment results and analytical results for fixator joint adjustment value. The errors between experiment results and analytic results are very small and within twenty percent, meaning that the experimental results can verify the correctness of the analysis results.

## 4. Discussion

In this paper, the sensitivity analyses of pin deviation on fracture dislocation and the influence of pin deviation on fixator joint adjustment were presented; these researches provide important information and rules to doctor and researchers.

For sensitivity analysis, firstly, when there is only one kind of pin deviation, pin deviation having the maximum sensitivity can be found. Although *φ*_1_, *φ*_2_, *φ*_3_ are all the orientation deviation and s_1_, s_2_, s_3_ are all the position deviation, the sensitivity are not the same. According to the value of the maximum sensitivity, for orientation deviation, it can be judged that the sensitivity of *φ*_2_ is greater than *φ*_3_ and *φ*_1_; the sensitivity of *φ*_3_ is larger than that of *φ*_1_. For position deviation, s_2_ has the highest sensitivity and s_1_ has the least sensitivity compared with s_2_. Comparing orientation deviation with position deviation, the sensitivity of orientation deviation is relatively large. This phenomenon explained in previous study that angle deformation was difficult to correct which need to regulate more fixator joints [19]. Secondly, when there were many kinds of deviations, the location of the maximum sensitivity can be found by seeking the maximum sensitivity in single deviation. This discovery will reduce a lot time of computation, and as long as we know the position of the maximum sensitivity of the single deviation, the location of maximum sensitivity can also be found for the multiple deviations.

Based on clinical case, the effects of pin deviation on fixator joint are also investigated. Firstly, for orientation deviation, *φ*_1_ have a great influence on fixator joint for a_8_. *φ*_2_ have a great influence on a_5_, d, and a_10_. *φ*_3_ have a great influence on a_6_ and a_9_. Through the comparison, the impact of *φ*_1_, *φ*_2_, and *φ*_3_ on fixator joints, due to *φ*_2_, affects more joints with more adjustment value and from this point of view, in which it can be drawn that *φ*_2_ has the biggest influence on the adjustment of fixator; the effect of *φ*_1_ is minimal. Meanwhile, for position deviation, s_3_ has a great influence on a_5_, a_8_, and a_10_. Due to the existence of s_2_, the regulatory value of a_9_ and a_6_ changed a lot. s_1_ made a great change in d. Comparing s_2_, s_1_, and s_3_, s_3_ can affect more joints, meaning when the deviation of s_3_ occurs, it is required to adjust more joints with greater amount of regulation in order to correct fracture completely; s_3_ has greater influence on the adjustment of fixator joint than s_1_ and s_2_. The impact of s_1_ is minimal. The results of the effects of pin deviation on fixator joints verify the sensitivity analysis from a point of view. The greater the sensitivity of pin deviation, the more the joints affected. Another interesting finding is that the impact of pin deviation on fixator joints has the property of superposition; that is, if any deviations exist at the same time, that will have an impact on more fixator joints, which are equal to the sum of the effect of each deviation on fixator joint adjustment. Meanwhile, based on the clinical case, seven situations of pin deviation in experimental were set up to verify the analytic results. Moreover, in clinical practice, at the ideal situation, the adjustment values of fixator joints are consistent with analytical results and experimental results

Through these analytic results from these findings, the orthopedist according to the actual situations reasonably increases or decreases the adjustment value of fixator joints, which could help correct the fracture residual dislocation caused by pin deviation; it also helps in reducing anesthesia time, lowering patient morbidity, and potentially lowering the risk of infection and radiation exposure, which also helps medical personnel to flexibly correct the fracture. The decrease of joint regulation due to the existence of pin deviation will increase the joint solution domain so as to expand the application scope of various bone deformities and avoid reaching the limit value of joints. Without knowing these information, complex reduction pattern could only be determined through trial and error, and pin needs to be inserted into bone more than once, which could prolong operation time and duplicate drilling attempts which may leave more bone tunnels that could potentially affect ligamentous reconstructions or complex fracture reduction [20].

The analysis technique and results reported herewith can provide useful information for the medical personnel who use external fixators in the management of long bone fractures and for device manufacturers to enhance their products' efficacy in adjustability, modify their current fixators, or develop new devices. These results should also be considered for possible manual or motorized adjustment in the next generation of external fixator.

## 5. Conclusions

Different pin deviation has different sensitivity; the greater the sensitivity of pin deviation, the greater the effect on the accuracy of fracture correction and the adjustment of fixator. The regulatory analysis and sensitivity analysis of pin deviation are validated by each other, based on a clinical case. The greater sensitivity of pin deviation means that it may be more likely to cause fracture dislocation and then need to adjust more joints. Moreover, the experiments are established to verify the analytic results. This research could provide the important theoretical foundation for orthopedics and these are crucial steps toward its successful applications.

## Figures and Tables

**Table 1:** The joint parameters of unilateral external fixator.

Joint variable	Kinematic pair	limit value of joints	Rotational axis
a_5_	Distal revolute pair	0~360 (degree)	Z_5_
a_6_	Distal revolute pair	0~120 (degree)	Z_6_
d	Moving pair	0~40(mm)	Z_7_
a_8_	Proximal revolute pair	0~360 (degree)	Z_8_
a_9_	Proximal revolute pair	0~120 (degree)	Z_9_
a_10_	Proximal revolute pair	0~360 (degree)	Z_10_

**Table 2:** The parameters of pin deviation for unilateral external fixator.

Symbol of pin deviations	pin deviations	Error range of angle deviation	Angle deviation axis
*φ*_1_	Distal angel deviation	0~20 (degree)	Z_1_
*φ*_2_	Distal angel deviation	0~20 (degree)	Z_2_
*φ*_3_	Distal angel deviation	0~20 (degree)	Z_3_
s_1_	Distal position deviation	0~10mm	Z_1_
s_2_	Distal position deviation	0~10mm	Z_2_
s_3_	Distal position deviation	0~10mm	Z_3_

**Table 3:** The corresponding amount of various types deviation of pins.

Types of pin deviation	Fracture dislocation	Deviation transfer matrix	Error model in fracture site
Δ*s*_1_	Δ*Bs*_1_	*Js*_1_	Δ*Bs*_1_ = *Js*_1_Δ*s*_1_
Δ*s*_2_	Δ*Bs*_2_	*Js*_2_	Δ*Bs*_2_ = *Js*_2_Δ*s*_2_
Δ*s*_3_	Δ*Bs*_3_	*Js*_3_	Δ*Bs*_3_ = *Js*_3_Δ*s*_3_
Δ*φ*_1_	Δ*Bφ*_1_	*Jφ*_1_	Δ*Bφ*_1_ = *Jφ*_1_Δ*φ*_1_
Δ*φ*_2_	Δ*Bφ*_2_	*Jφ*_2_	Δ*Bφ*_2_ = *Jφ*_2_Δ*φ*_2_
Δ*φ*_3_	Δ*Bφ*_3_	*Jφ*_3_	Δ*Bφ*_3_ = *Jφ*_3_Δ*φ*_3_

**Table 4:** The adjustment values of fixator joints at seven situations for experimental results.

Four situations	a_5_(degree)	a_6_(degree)	d(mm)	a_8_(degree)	a_9_(degree)	a_10_(degree)
Ideal situation	-5.5	20	9	-10	-3	-12
*φ*_1_=20°	0.5	8.5	3	4	-9.5	-9
*φ*_2_=20°	16	21	-8	-16.5	-6.5	-20.5
*φ*_3_=20°	5.5	-5	7.5	-14	2.5	-9.3
s_1_=10mm	-5	18.5	18.5	-11.5	-1.2	-10.5
s_2_=10mm	-5.6	15	6.0	-12	2	-8.5
s_3_=10mm	-12	20	10.5	-6.5	-4.5	-5

**Table 5:** The errors between experimental and analytical results for fixator joint adjustment values.

Four situations	a_5_(degree)	a_6_(degree)	d(mm)	a_8_(degree)	a_9_(degree)	a_10_(degree)
Ideal situation	8%	3.8%	3.78%	5.2%	8.27%	1.05%
*φ*_1_=20°	16%	2.96%	13.4%	7.69%	1.56%	1.26%
*φ*_2_=20°	4.7%	0.213%	3.12%	1.17%	1%	1.7%
*φ*_3_=20°	1.7%	1.82%	5.4%	3.05%	0.56%	19.19%
s_1_=10mm	7.4%	2.32%	1.14%	5.41%	9.7%	0.52%
s_2_=10mm	5%	1.51%	4.11%	9.33%	3.7%	5.357%
s_3_=10mm	0.35%	2.43%	3%	19.7%	3.17%	0.85%

## Data Availability

The data used in this study are analyzed and experimented by ourselves

## Appendix

First, according to (9), (A.1) can be solved as follows:

(A.1) $$\begin{array}{c} d_{7}o_{1x}\sin\left(a_{6}\right)+d_{7}n_{1x}\cos\left(a_{5}\right)\cos\left(a_{6}\right)+d_{7}a_{1x}\sin\left(a_{5}\right)\cos\left(a_{6}\right)=E\\ d_{7}o_{1y}\sin\left(a_{6}\right)+d_{7}n_{1y}\cos\left(a_{5}\right)\cos\left(a_{6}\right)+d_{7}a_{1y}\sin\left(a_{5}\right)\cos\left(a_{6}\right)=F\\ d_{7}o_{1z}\sin\left(a_{6}\right)+d_{7}n_{1z}\cos\left(a_{5}\right)\cos\left(a_{6}\right)+d_{7}a_{1z}\sin\left(a_{5}\right)\cos\left(a_{6}\right)=G \end{array}$$

According to (A.1), the fixator joint values—a_5_  *a*_6_ d —can be obtained by (A.2). Detailed solution process can be shown as follows:

(A.2) $$\begin{array}{c} a_{5}=2\mathrm{asin}\left(\frac{2U_{5}}{\left(1+U_{5}^{2}\right)}\right)\\ a_{6}=2\mathrm{atan}\left(\frac{\left(-R_{7}-\sqrt{{\left(R_{7}\right)}^{2}+{\left(R_{8}\right)}^{2}}\right)}{-R_{8}}\right)\\ a_{7}=\frac{E}{\left(o_{1x}J+n_{1x}JW+a_{1x}WJ\right)}\\ d=d_{7}-D \end{array}$$

Second, solving fixator joints of a_10_, a_9_, and a_8_. According to (15), (A.3) can be obtained:

(A.3) $$\begin{array}{c} S_{1}=N_{1}\sin\left(a_{9}\right)+N_{2}\cos\left(a_{9}\right)\cos\left(a_{10}\right)+N_{3}\cos\left(a_{9}\right)\sin\left(a_{10}\right)\\ S_{2}=N_{4}\sin\left(a_{9}\right)+N_{5}\cos\left(a_{9}\right)\cos\left(a_{10}\right)+N_{6}\cos\left(a_{9}\right)\sin\left(a_{10}\right)\\ S_{3}=N_{7}\sin\left(a_{9}\right)+N_{8}\cos\left(a_{9}\right)\cos\left(a_{10}\right)+N_{9}\cos\left(a_{9}\right)\sin\left(a_{10}\right) \end{array}$$

According to (A.3), a_10_, a_9_, and a_8_ can be obtained by

(A.4) $$\begin{array}{c} a_{10}=2\mathrm{atan}\frac{-N_{17}+\sqrt{N_{17}^{2}-N_{16}N_{18}}}{N_{16}}\\ a_{9}=2\mathrm{atan}\frac{-N_{1}+\sqrt{N_{1}^{2}+N_{19}^{2}-N_{16}N_{18}-S_{1}^{2}}}{-N_{19}-S_{1}}\\ a_{8}=2\mathrm{atan}\frac{-Q_{12}+\sqrt{Q_{12}^{2}+Q_{11}^{2}-Q_{13}^{2}}}{Q_{11}+Q_{13}} \end{array}$$

In (10), (15), and (18), the detailed expression of A_1_ ~A_8_, B_1_ ~B_10_, C_1_ ~C_8_, D_1_ ~D_8_, E_1_ ~E_10_, and F_1_ ~F_8_ is shown as follows:

(A.5) $$\begin{array}{c} A_{1}=\cos\left(\varphi_{2}\right)\left(l_{1}+s_{3}\right)\left(-\sin\left(\varphi_{1}\right)\right)-s_{2}\cos\left(\varphi_{1}\right)-l_{2}\cos\left(\varphi_{1}\right)\sin\left(\varphi_{3}\right)-\sin\left(\varphi_{1}\right)\cos\left(\varphi_{3}\right)\sin\left(\varphi_{2}\right);\\ A_{2}=-\cos\left(\varphi_{1}\right)\sin\left(\varphi_{2}\right)\left(l_{1}+s_{3}\right)+\cos\left(\varphi_{1}\right)\cos\left(\varphi_{3}\right)\cos\left(\varphi_{2}\right);\\ A_{3}=-l_{2}\sin\left(\varphi_{1}\right)\cos\left(\varphi_{3}\right)-\cos\left(\varphi_{1}\right)\sin\left(\varphi_{3}\right)\sin\left(\varphi_{2}\right);\\ A_{4}=\cos\left(\varphi_{1}\right)\cos\left(\varphi_{2}\right);\\ A_{5}=\sin\left(\varphi_{1}\right);\\ A_{6}=\sin\left(q\varphi_{2}\right);\\ A_{7}={Bs}_{3}\cos\left(q\varphi_{2}\right)+\cos\left(q\varphi_{2}\right)\left(l_{3}-l_{5}\right)-l_{4}\sin\left(q\varphi_{2}\right)\cos\left(q\varphi_{3}\right);\\ A_{8}=l_{4}\cos\left(q\varphi_{2}\right)\sin\left(q\varphi_{3}\right);\\ B_{1}=-l_{2}\sin\left(\varphi_{1}\right)\sin\left(\varphi_{3}\right)-l_{2}\cos\left(\varphi_{3}\right)\cos\left(\varphi_{1}\right)\sin\left(\varphi_{2}\right)-s_{2}\sin\left(\varphi_{1}\right)+\cos\left(\varphi_{2}\right)\sin\left(\varphi_{1}\right)\left(l_{1}+s_{3}\right);\\ B_{2}=-l_{2}\sin\left(\varphi_{1}\right)\cos\left(\varphi_{2}\right)\cos\left(\varphi_{3}\right)-\left(l_{1}+s_{3}\right)\sin\left(\varphi_{1}\right)\sin\left(\varphi_{2}\right);\\ B_{3}=l_{2}\cos\left(\varphi_{1}\right)\cos\left(\varphi_{3}\right)+l_{2}\sin\left(\varphi_{1}\right)\sin\left(\varphi_{3}\right)\sin\left(\varphi_{2}\right);\\ B_{4}=\cos\left(\varphi_{1}\right);\\ B_{5}=\cos\left(\varphi_{2}\right)\sin\left(\varphi_{1}\right);\\ B_{6}=-l_{4}\sin\left(q\varphi_{1}\right)\sin\left(q\varphi_{3}\right)+l_{4}\cos\left(q\varphi_{3}\right)\cos\left(q\varphi_{1}\right)\sin\left(q\varphi_{2}\right)-{Bs}_{2}\sin\left(q\varphi_{1}\right)-{Bs}_{3}\cos\left(q\varphi_{2}\right)\cos\left(q\varphi_{1}\right)-\cos\left(q\varphi_{2}\right)\cos\left(q\varphi_{1}\right)\left(l_{3}-l_{5}\right);\\ B_{7}=l_{4}\cos\left(q\varphi_{3}\right)\sin\left(q\varphi_{1}\right)\cos\left(q\varphi_{2}\right)+{Bs}_{3}\sin\left(q\varphi_{1}\right)\sin\left(q\varphi_{2}\right)+\sin\left(q\varphi_{2}\right)\sin\left(q\varphi_{1}\right)\left(l_{3}-l_{5}\right);\\ B_{8}=l_{4}\cos\left(q\varphi_{1}\right)\cos\left(q\varphi_{3}\right)-l_{4}\sin\left(q\varphi_{3}\right)\sin\left(q\varphi_{1}\right)\sin\left(q\varphi_{2}\right);\\ B_{9}=\cos\left(q\varphi_{1}\right);\\ B_{10}=\sin\left(q\varphi_{1}\right)\cos\left(q\varphi_{2}\right);\\ C_{1}=\cos\left(\varphi_{2}\right)\left(l_{1}+s_{3}\right)-l_{2}\sin\left(\varphi_{2}\right)\cos\left(\varphi_{3}\right);\\ C_{2}=l_{2}\cos\left(\varphi_{2}\right)\sin\left(\varphi_{3}\right);\\ C_{3}=\sin\left(\varphi_{2}\right);\\ C_{4}=l_{4}\cos\left(q\varphi_{1}\right)\sin\left(q\varphi_{3}\right)+l_{4}\sin\left(q\varphi_{1}\right)\cos\left(q\varphi_{3}\right)\sin\left(q\varphi_{2}\right)+{Bs}_{2}\cos\left(q\varphi_{1}\right)-{Bs}_{3}\sin\left(q\varphi_{1}\right)\cos\left(q\varphi_{2}\right)-\sin\left(q\varphi_{1}\right)\cos\left(q\varphi_{2}\right)\left(l_{3}-l_{5}\right);\\ C_{5}=l_{4}\cos\left(q\varphi_{1}\right)\cos\left(q\varphi_{3}\right)\cos\left(q\varphi_{2}\right)+{Bs}_{3}\cos\left(q\varphi_{1}\right)\sin\left(q\varphi_{2}\right)+\cos\left(q\varphi_{1}\right)\sin\left(q\varphi_{2}\right)\left(l_{3}-l_{5}\right);\\ C_{6}=l_{4}\sin\left(q\varphi_{1}\right)\cos\left(q\varphi_{3}\right)+l_{4}\cos\left(q\varphi_{1}\right)\sin\left(q\varphi_{3}\right)\sin\left(q\varphi_{2}\right);\\ C_{7}=\sin\left(q\varphi_{1}\right);\\ C_{8}=\cos\left(q\varphi_{1}\right)\cos\left(q\varphi_{2}\right);\\ D_{1}={Bs}_{3}\cos\left(q\varphi_{2}\right)+\cos\left(q\varphi_{2}\right)\left(l_{3}-l_{5}\right)-l_{4}\sin\left(q\varphi_{2}\right)\cos\left(q\varphi_{3}\right);\\ D_{2}=-l_{4}\cos\left(q\varphi_{2}\right)\sin\left(q\varphi_{3}\right);\\ D_{3}=\sin\left(q\varphi_{2}\right);\\ D_{4}=-\sin\left(\varphi_{1}\right)\cos\left(\varphi_{2}\right)\left(l_{1}+s_{3}\right)-s_{2}\cos\left(\varphi_{1}\right)-l_{2}\cos\left(\varphi_{1}\right)\sin\left(\varphi_{3}\right)-d_{7}\sin\left(a_{6}\right)\cos\left(\varphi_{3}\right)\cos\left(\varphi_{1}\right)+l_{2}\sin\left(\varphi_{1}\right)\cos\left(\varphi_{3}\right)\sin\left(\varphi_{2}\right)-d_{7}\cos\left(a_{6}\right)\sin\left(a_{5}\right)\sin\left(\varphi_{1}\right)\cos\left(\varphi_{2}\right)-d_{7}\cos\left(a_{6}\right)\cos\left(a_{5}\right)\cos\left(\varphi_{1}\right)\sin\left(\varphi_{3}\right)-d_{7}\sin\left(a_{6}\right)\sin\left(\varphi_{1}\right)\sin\left(\varphi_{2}\right)\sin\left(\varphi_{3}\right)+d_{7}\cos\left(a_{6}\right)\cos\left(a_{5}\right)\sin\left(\varphi_{1}\right)\sin\left(\varphi_{2}\right)\cos\left(\varphi_{3}\right);\\ D_{5}=-\sin\left(\varphi_{2}\right)\cos\left(\varphi_{1}\right)\left(l_{1}+s_{3}\right)-l_{2}\cos\left(\varphi_{1}\right)\cos\left(\varphi_{3}\right)\cos\left(\varphi_{2}\right)-d_{7}\cos\left(a_{6}\right)\sin\left(a_{5}\right)\cos\left(\varphi_{1}\right)\sin\left(\varphi_{2}\right)+d_{7}\sin\left(a_{6}\right)\cos\left(\varphi_{2}\right)\cos\left(\varphi_{1}\right)\sin\left(\varphi_{3}\right)-d_{7}\cos\left(a_{6}\right)\cos\left(a_{5}\right)\cos\left(\varphi_{1}\right)\cos\left(\varphi_{2}\right)\cos\left(\varphi_{3}\right);\\ D_{6}=-l_{2}\sin\left(\varphi_{1}\right)\cos\left(\varphi_{3}\right)+d_{7}\sin\left(a_{6}\right)\sin\left(\varphi_{1}\right)\sin\left(\varphi_{3}\right)+l_{2}\cos\left(\varphi_{1}\right)\sin\left(\varphi_{3}\right)\sin\left(\varphi_{2}\right)-d_{7}\cos\left(a_{6}\right)\cos\left(a_{5}\right)\sin\left(\varphi_{1}\right)\cos\left(\varphi_{3}\right)+d_{7}\sin\left(a_{6}\right)\cos\left(\varphi_{1}\right)\sin\left(\varphi_{2}\right)\cos\left(\varphi_{3}\right)+d_{7}\cos\left(a_{6}\right)\cos\left(a_{5}\right)\cos\left(\varphi_{1}\right)\sin\left(\varphi_{2}\right)\sin\left(\varphi_{3}\right);\\ D_{7}=\sin\left(\varphi_{1}\right);\\ D_{8}=\cos\left(\varphi_{1}\right)\cos\left(\varphi_{2}\right);\\ E_{1}=-l_{4}\sin\left(q\varphi_{1}\right)\sin\left(q\varphi_{3}\right)+l_{4}\cos\left(q\varphi_{3}\right)\cos\left(q\varphi_{1}\right)\sin\left(q\varphi_{2}\right)-{Bs}_{2}\sin\left(q\varphi_{1}\right)-{Bs}_{3}\cos\left(q\varphi_{2}\right)\cos\left(q\varphi_{1}\right)-\cos\left(q\varphi_{2}\right)\cos\left(q\varphi_{1}\right)\left(l_{3}-l_{5}\right);\\ E_{2}=l_{4}\cos\left(q\varphi_{3}\right)\sin\left(q\varphi_{1}\right)\cos\left(q\varphi_{2}\right)+{Bs}_{3}\sin\left(q\varphi_{2}\right)\sin\left(q\varphi_{1}\right)+\sin\left(q\varphi_{2}\right)\sin\left(q\varphi_{1}\right)\left(l_{3}-l_{5}\right);\\ E_{3}=l_{4}\cos\left(q\varphi_{3}\right)\cos\left(q\varphi_{1}\right)-l_{4}\sin\left(q\varphi_{2}\right)\sin\left(q\varphi_{1}\right)\sin\left(q\varphi_{3}\right);\\ E_{4}=\cos\left(q\varphi_{1}\right);\\ E_{5}=\cos\left(q\varphi_{2}\right)\sin\left(q\varphi_{1}\right);\\ E_{6}=-s_{2}\sin\left(\varphi_{1}\right)+l_{1}\cos\left(\varphi_{2}\right)\cos\left(\varphi_{1}\right)-l_{2}\sin\left(\varphi_{1}\right)\sin\left(\varphi_{3}\right)+s_{3}\cos\left(\varphi_{2}\right)\cos\left(\varphi_{1}\right)-d_{7}\sin\left(a_{6}\right)\sin\left(\varphi_{1}\right)\cos\left(\varphi_{3}\right)-l_{2}\cos\left(\varphi_{3}\right)\cos\left(\varphi_{1}\right)\sin\left(\varphi_{2}\right)-d_{7}\cos\left(a_{6}\right)\cos\left(a_{5}\right)\sin\left(\varphi_{1}\right)\sin\left(\varphi_{3}\right)+d_{7}\cos\left(a_{6}\right)\sin\left(a_{5}\right)\cos\left(\varphi_{1}\right)\cos\left(\varphi_{2}\right)+d_{7}\sin\left(a_{6}\right)\cos\left(\varphi_{1}\right)\sin\left(\varphi_{2}\right)\sin\left(\varphi_{3}\right)-d_{7}\cos\left(a_{6}\right)\cos\left(a_{5}\right)\cos\left(\varphi_{1}\right)\sin\left(\varphi_{2}\right)\cos\left(\varphi_{3}\right);\\ E_{7}=l_{1}\sin\left(\varphi_{2}\right)\sin\left(\varphi_{1}\right)+s_{3}\sin\left(\varphi_{2}\right)\sin\left(\varphi_{1}\right)+l_{2}\cos\left(\varphi_{2}\right)\cos\left(\varphi_{3}\right)\sin\left(\varphi_{1}\right)+d_{7}\cos\left(a_{6}\right)\sin\left(a_{5}\right)\sin\left(\varphi_{1}\right)\sin\left(\varphi_{2}\right)-d_{7}\sin\left(a_{6}\right)\cos\left(\varphi_{2}\right)\sin\left(\varphi_{1}\right)\sin\left(\varphi_{3}\right)+d_{7}\cos\left(a_{6}\right)\cos\left(a_{5}\right)\cos\left(\varphi_{2}\right)\sin\left(\varphi_{1}\right)\cos\left(\varphi_{3}\right);\\ E_{8}=l_{2}\cos\left(\varphi_{1}\right)\cos\left(\varphi_{3}\right)-d_{7}\sin\left(a_{6}\right)\cos\left(\varphi_{1}\right)\sin\left(\varphi_{3}\right)+l_{2}\sin\left(\varphi_{3}\right)\sin\left(\varphi_{1}\right)\sin\left(\varphi_{2}\right)+d_{7}\cos\left(a_{6}\right)\cos\left(a_{5}\right)\cos\left(\varphi_{1}\right)\cos\left(\varphi_{3}\right)+d_{7}\sin\left(a_{6}\right)\cos\left(\varphi_{3}\right)\sin\left(\varphi_{2}\right)\sin\left(\varphi_{1}\right)+d_{7}\cos\left(a_{6}\right)\cos\left(a_{5}\right)\sin\left(\varphi_{1}\right)\sin\left(\varphi_{2}\right)\sin\left(\varphi_{3}\right);\\ E_{9}=\cos\left(\varphi_{1}\right);\\ E_{10}=\cos\left(\varphi_{2}\right)\sin\left(\varphi_{1}\right);\\ F_{1}=l_{4}\cos\left(q\varphi_{1}\right)\sin\left(q\varphi_{3}\right)+l_{4}\sin\left(q\varphi_{1}\right)\cos\left(q\varphi_{3}\right)\sin\left(q\varphi_{2}\right)+{Bs}_{2}\cos\left(q\varphi_{1}\right)-{Bs}_{3}\sin\left(q\varphi_{1}\right)\cos\left(q\varphi_{2}\right)-\sin\left(q\varphi_{1}\right)\cos\left(q\varphi_{2}\right)\left(l_{3}-l_{5}\right);\\ F_{2}=l_{4}\cos\left(q\varphi_{1}\right)\cos\left(q\varphi_{3}\right)\cos\left(q\varphi_{2}\right)+{Bs}_{3}\cos\left(q\varphi_{1}\right)\sin\left(q\varphi_{2}\right)+\sin\left(q\varphi_{2}\right)\cos\left(q\varphi_{1}\right)\left(l_{3}-l_{5}\right);\\ F_{3}=l_{4}\sin\left(q\varphi_{1}\right)\cos\left(q\varphi_{3}\right)+l_{4}\sin\left(q\varphi_{2}\right)\cos\left(q\varphi_{1}\right)\sin\left(q\varphi_{3}\right);\\ F_{4}=\sin\left(q\varphi_{1}\right);\\ F_{5}=\cos\left(q\varphi_{1}\right)\cos\left(q\varphi_{2}\right);\\ F_{6}=\cos\left(\varphi_{2}\right)l_{1}+s_{3}\cos\left(\varphi_{2}\right)-l_{2}\sin\left(\varphi_{2}\right)\cos\left(\varphi_{3}\right)+d_{7}\cos\left(a_{6}\right)\sin\left(a_{5}\right)\cos\left(\varphi_{2}\right)+d_{7}\sin\left(a_{6}\right)\sin\left(\varphi_{2}\right)\sin\left(\varphi_{3}\right)-d_{7}\cos\left(a_{6}\right)\cos\left(a_{5}\right)\cos\left(\varphi_{3}\right)\sin\left(\varphi_{2}\right);\\ F_{7}=l_{2}\cos\left(\varphi_{2}\right)\sin\left(\varphi_{3}\right)+d_{7}\sin\left(a_{6}\right)\cos\left(\varphi_{2}\right)\cos\left(\varphi_{3}\right)+d_{7}\cos\left(a_{6}\right)\cos\left(a_{5}\right)\cos\left(\varphi_{2}\right)\sin\left(\varphi_{3}\right);\\ F_{8}=\sin\left(\varphi_{2}\right); \end{array}$$

## References

[1] Silva F. G, Moura M. F. S. F. D., Dourado N. (2017). Fracture characterization of human cortical bone under mode II loading using the end-notched flexure test. *Medical & Biological Engineering & Computing*.

[2] Egger E. L., Gottsauner-Wolf F., Palmer J., Aro H. T., Chao E. Y. S. (1993). Effects of axial dynamization on bone healing. *Journal of Trauma*.

[3] Goodship A. E., Kenwright J. (1985). The influence of induced micromovement upon the healing of experimental tibial fractures. *The Journal of Bone & Joint Surgery*.

[4] Wolf S., Augat P., Eckert-Hübner K., Laule A., Krischak G. D., Claes L. E. (2001). Effects of high-frequency, low-magnitude mechanical stimulus on bone healing. *Clinical Orthopaedics and Related Research*.

[5] Cooke T. D. V., Pichora D., Siu D., Scudamore R. A., Bryant J. T. (1989). Surgical implications of varus deformity of the knee with obliquity of joint surfaces. *The Journal of Bone & Joint Surgery*.

[6] Paley D., Chaudray M., Pirone A. M., Lentz P., Kautz D. (1990). Treatment of malunions and mal-nonunions of the femur and tibia by detailed preoperative planning and the Ilizarov techniques. *Orthopedic Clinics of North America*.

[7] Rezvani-Sharif A., Tafazzoli-Shadpour M., Kazemi-Saleh D., Sotoudeh-Anvari M. (2017). Stress analysis of fracture of atherosclerotic plaques: crack propagation modeling. *Medical & Biological Engineering & Computing*.

[8] Kanel J. S., Price C. T. (1995). Unilateral external fixation for corrective osteotomies in patients with hypophosphatemic rickets. *Journal of Pediatric Orthopaedics*.

[9] Chao E. Y. S., Rim K., Smidt G. L., Johnston R. C. (1970). The application of 4 × 4 matrix method to the correction of the measurements of hip joint rotations. *Journal of Biomechanics*.

[10] Aro H. T., Chao E. Y. S. (1993). Biomechanics and biology of fracture repair under external fixation. *Hand Clinics*.

[11] Raymond W. (2005). Computational simulation of axial dynamization on long bone fractures. *Journal of Clinical Biomechanics*.

[12] Paley D., Tetsworth K. (1992). Mechanical axis deviation of the lower limbs: Preoperative planning of uniapical angular deformities of the tibia or femur. *Clinical Orthopaedics and Related Research*.

[13] Chen G., Wu F. Y., Zhang J. Q., Zhong G. Q., Liu F. (2015). Sensitivities of biomechanical assessment methods for fracture healing of long bones. *Medical Engineering & Physics*.

[14] Kim Y. H., Inoue N., Chao E. Y. S. (2002). Kinematic simulation of fracture reduction and bone deformity correction under unilateral external fixation. *Journal of Biomechanics*.

[15] Koo T. K. K., Chao E. Y. S., Mak A. F. T. (2006). Development and validation of a new approach for computer-aided long bone fracture reduction using unilateral external fixator. *Journal of Biomechanics*.

[16] Liu R. W., Kim Y. H., Lee D. C., Inoue N., Koo T. K., Chao E. Y. S. (2005). Computational simulation of axial dynamization on long bone fractures. *Clinical Biomechanics*.

[17] Ou Y.-J. (2009). Kinematic adjustability of unilateral external fixators for fracture reduction and alignment of axial dynamization. *Journal of Biomechanics*.

[18] Wang W., Yun C. (2009). Orthogonal experimental design to synthesize the accuracy of robotic mechanism. *Journal of Mechanical Engineering*.

[19] Paley D. (2002). *Principles of Deformity Correction*.

[20] Deuel C. R., Wolinsky P., Shepherd E., Hazelwood S. J. (2007). The use of hinged external fixation to provide additional stabilization for fractures of the distal humerus. *Journal of Orthopaedic Trauma*.

